# Exploring the role of environmental pollution and trade openness in human health in the context of sustainable development

**DOI:** 10.1371/journal.pone.0312246

**Published:** 2025-03-10

**Authors:** Zhenzhi Lu, Lin Jiang, Xinyue Wang

**Affiliations:** 1 The Affiliated Stomatological Hospital of Jiujiang University, Jiujiang, Jiangxi, China,; 2 Department of Trade and Management, Woosuk University, Jeonju, Jeollabuk-do, Korea,; 3 Shandong Women’s University, Jinan, China; Flinders University, AUSTRALIA

## Abstract

Carbon-neutral development can significantly reduce the concentration of pollutants in the atmosphere and the occurrence of human health problems through the use of clean energy and promotion of energy efficiency. Both environmental pollution and trade openness are important factors that affect human health, and this paper verifies the relationship between the three by using systematic GMM modeling. The following conclusions are drawn: (1) At the national level, although trade openness inhibits human health, this effect is not significant. From the perspective of different regions, trade openness can enhance public health in the eastern region but is unfavorable to human health in the central and western regions. (2) Environmental pollution reduces the human health level in all regions; however, it is not significant in the eastern region, which is related to the high proportion of clean energy, and the central and western regions are mainly dominated by and overly dependent on the energy industry, thus causing serious negative impacts on the environment, which is not conducive to human health. (3) Urbanization and human health show a significant and homogeneous relationship in the national and eastern samples, fail the test of significance in the central region, and have a lower level of significance in the western region. Increases in public health expenditures reduce population mortality, and the effect is significant in all regions. Increasing population size has a significant dampening effect on human health at the national level and in the western and central regions, but there is a positive ameliorating effect in the eastern region. Environmental regulatory policies can be effective in reducing population mortality in all regions, thus enhancing human health.

## Introduction

In the face of the climate crisis, China has put forward the goal of carbon neutrality. Peak carbon and carbon-neutral processes are in harmony with the goals of China’s health system, which is committed to providing high-quality health protection for its people. Human health is the basic yardstick for measuring people’s happiness, and human health is a proper connotation and an important part of the comprehensive construction of a modern socialist country [1–2]. Along with the official implementation of the “Healthy China 2030” planning program, human issues have become the focus of attention for the whole population. In June 2019, China put forward actions to guide the prevention of disease and health promotion over the next decade. A series of major strategic deployments have broken the action guidelines for the future development of human health. In recent years, as China’s economy and society have surpassed a series of major development achievements, the people’s attention to health and safety and quality of life has been increasing, while the development problems left over from the previous stages (such as environmental pollution and ecological damage) are still threatening human health, and the risk of international pandemics such as Influenza A, Ebola hemorrhagic fever, and neo-coronavirus pneumonia is also treacherous. Human health issues are therefore of great concern to all sectors of society [3–4].

As global trade increases, there is more frequent movement of people and goods, which may increase the risk of disease transmission. For example, new pathogens may be introduced into new areas through international trade channels, thereby increasing the potential for disease transmission. This may pose a threat to public health, especially in the absence of effective prevention and control measures. In addition, globalization has facilitated the flow of industrial products across borders, but this may also lead to increased environmental pollution problems [5–8]. For example, the large amount of waste and pollutants that may be generated during certain industrial production processes, if not effectively dealt with, may have a negative impact on the environment, including air and water pollution, which, in turn, may affect human health problems. With the continuous expansion of the scale of import and export trade, international trade has become increasingly important to China’s economy, and its impact on human health has become more and more prominent, becoming an important driving force for China’s global health strategy and the construction of a healthy China. At the same time, as foreign trade continues to open up, national health as a kind of human capital is also receiving more and more attention from the Chinese government; health, as a basic right and demand of residents, is one of the most basic needs of human beings and also one of the basic dimensions that can be used to measure human development. In the new period of China’s domestic and international double-cycle pattern construction with a background of high-level opening up, international trade and the health of China’s residents being more closely linked to the public will further highlight the health effects, and clarifying the link between the two has become more important. Research on international trade and residents’ health has also gained the attention of many scholars and, in the current context, has more important theoretical significance and practical significance.

Meanwhile, according to the World Health Organization, 90% of the world’s population breathes polluted air every day. Air pollution contributes to the early deaths of 7 million people each year from diseases such as cancer, stroke, heart disease, and lung disease. A total of 90% of those premature deaths occur in low- and middle-income countries with high emissions of pollutants from industry, transport, and agriculture, where household use of unclean cookstoves and fuels is also higher. Fossil fuel combustion is a major contributor to climate change and has significant health impacts. Climate change is projected to cause 250,000 deaths per year globally between 2030 and 2050, causing malnutrition, malaria, diarrhea, and heat stress. Since the beginning of reform and opening up, China’s per capita GDP has been growing rapidly, and in 2021, the per capita consumption expenditure of national residents was CNY 24,100, of which per capita healthcare consumption expenditure was CNY 2,115, accounting for 8.8% of per capita consumption expenditure, an increase of 14.8% year-on-year. However, with the rapid development of the economy, the phenomenon of environmental pollution in China is becoming more and more serious. Haze weather occurs in many places in China, and severe haze weather can cause great safety hazards to public transportation and can also affect the efficiency of urban commuting, leading to an increase in transportation costs and a decline in social and economic benefits [9–10]. In addition, due to the complex composition of haze, which often contains a variety of metal substances, these fine particles will attach to electric locomotives, which might easily lead to the phenomenon of “dirty flash”, causing impacts to tram and railroad networks. Haze weather can lead to restrictions on air transportation due to the reduced visibility of the atmosphere, which leads to a large number of flights being delayed and customer goods not being delivered in a timely manner, indirectly causing economic losses. Severe haze pollution will also have an impact on agriculture, and is not conducive to the growth of plants. The health risk to residents caused by environmental pollution has become a worldwide topic, and it is even more serious in some countries and regions where the provision of public services is relatively insufficient [11–12]. The above situation makes us think about the following questions: what is the correlation between the aggravation of environmental pollution, continuous economic growth, the improvement of health services, and human health risks caused by environmental pollution in China? Given the differences in the level of economic development of the different regions of China, what about human health in different regions? The discussion of these questions will provide an important reference for central and local governments to formulate targeted policies for the sustainable development of environmental pollution and human health.

Therefore, in the context of realizing the goals of “Healthy China” and “Double Carbon”, we studied the relationship between environmental pollution, trade openness, and human health, which represents crossdisciplinary considerations across health science and economics. There are many international studies on this issue, but there are fewer domestic studies on China’s problems. Therefore, the main contributions of this thesis are as follows: firstly, carrying out research by incorporating the three main aspects into the same framework can fill the gaps in existing research and improve and supplement it; secondly, based on China’s national conditions and exploring the green development strategies of countries with environmental and health problems in the context of sustainable development, the policy recommendations derived from this thesis can provide a theoretical reference for governmental policy formulation. We chose the interprovincial panel macro data of 30 regions in China to study this issue; additionally, based on combing the publicly available data in China, we selected 30 regional datasets in China from 2008 to 2022 to conduct an empirical study based on the availability and completeness of the data and conducted comparative and econometric analyses of the relationship between environmental pollution, trade openness, and human health; this aims to make clear the relationships between the two on human health, which, to a certain extent, compensates for the insufficiency of the study on the issue. At the same time, it is hoped that it will provide a reference for the country to formulate energy policy and health and wellness policy.

## Literature review

### Research on human health

At present, domestic and foreign academic research on human health has achieved rich results, and the research content closely related to this paper can be summarized in three aspects:

Firstly, research on the theoretical connotation of human health. Initially, health is mainly interpreted from the perspective of bodily functions, referring to physiological health, which belongs to a narrow concept of health [13]. Health refers to the absence of physical disease and a better state of mental and social adaptation, in addition to moral health, on top of which there are 10 other criteria that are widely recognized in society, including a strong appetite, strong resilience, and so on. Among them, the physical aspect mainly refers to the human body being healthy, including no disability and disease, nutritional standards, etc. Our research was carried out from the macro level, and mainly focuses on physical health and does not consider psychological, social, and moral factors.

Secondly, research about the measurement of human health is considered. The current measurement methods on human health can be broadly categorized into three types: (1) Using individual representative indicators to characterize human health. Zhang et al. [14] used the medical total health costs as a negative measure of human health; Xing and Tian [15] chose the number of lung cancer deaths, the number of oncology outpatient emergencies, and the number of new lung cancer cases to negatively characterize urban human health. (2) The efficiency values of multiple inputs and multiple outputs are measured. Wang and Liang [16] measured the efficiency of urban government health expenditure in China using Bootstrap-DEA and window DEA analysis with per capita budgeted fiscal health expenditure as the input variable and medical institutions per capita, beds per capita, and health technicians per capita as the output variables. Yu et al. [17] used the DEA-Malmquist model to dynamically measure the productivity of residents’ health in China’s eight comprehensive economic zones. (3) There is also some literature that constructs a comprehensive indicator system related to health measurement. Wang [18] used the weighted average of life expectancy, birth rate, and death rate to construct a health level index for each region in China as a reflection of the effect of health investment in each region. Zhao et al. [19] constructed an evaluation index system for healthy cities from four aspects, namely, health dimensions, health services, environmental dimensions, and socio-economic dimensions, based on the existing studies.

Thirdly, research on the factors that influence human health is also considered. Schultz [20] emphasized that health is an important component of human capital. Subsequently, many scholars have conducted extensive research on the importance of healthy human capital to economic growth; Fogel [21] pointed out that an improvement in food consumption could improve individual health. In recent years, a few scholars have conducted research on the causal identification between the two, including by taking a single region as the object of research and using the Grossman function model to study air pollutants’ impact on health [22]. Some scholars use panel data and econometric modeling to study the impact of air quality and other elements on human health [23] and make a comparative analysis of the differences between men and women, as well as the differences between the health effects of residents in developed and developing countries [24]. Staff Mestl [25] used Monte Carlo simulation to study the impact of indoor air pollution produced by domestic fuel combustion on the health of residents in Shanxi Province.

### Trade openness impact on human health

Existing research suggests that trade openness affects human health through food. For example, Ravuvu et al. [26] found that trade significantly increased the number of healthy food products, thereby promoting health. There are also some scholars who are opposed to this; Geof et al. [27] argued that trade liberalization, although only to a certain extent, provides food for poor countries to lift them out of poverty, but it may also make other countries over-nutritional through the transmission of unhealthy diets, thus adversely affecting their health.

Second, trade openness leads to technological spillovers that promote medical innovation and improve healthcare. For example, Papageorgiou et al. [28] found that trade openness facilitates medical technology spillovers, thereby improving human health. Kshetri and Dholakia [29] found that trade improves the level of healthcare services in a country and promotes their diversification, which effectively improves the quality of health of the local population. In addition, the system of trade in healthcare services guarantees the safety and reliability of the distribution of medicines to the population, which protects the fairness of the healthcare services. Li et al. [30] found that the expansion of trade not only improves medical services but also helps to enhance the demand for them among China’s residents, which, in turn, improves the health of China’s residents.

Third, trade openness affects human health through the environment. Scholars generally agree that environmental pollution can harm the health status of residents [31–32]. From the perspective of trade, a portion of scholars believe that international trade will bring cleaner production technology to a country, which reduces the country’s pollutant emissions, thus improving the living environment and health quality of its residents; for example, through the study of US data, Levinson [33] found that trade brings technological innovation, which reduces the pollution status of the environment and improves the level of health. Zhang [34] explored the impact of trade openness on the health of the population and found that international trade does not statistically significantly effect environmental pollution, and therefore, international trade does not affect the health of the population through environmental effects. However, some scholars believe that international trade will bring more serious environmental pollution, which, in turn, will harm residents’ health. Chen et al. [35] used a panel of 31 provinces and cities from 2000 to 2013 and found that international trade reduces residents’ health through the channel of environmental pollution. Bontems and Gozlan [36] similarly found that the environmental pollution caused by international trade worsens the health of low-income classes. Lin [37] explored trade liberalization’s impact on the health of China’s residents and found that trade openness exacerbated environmental pollution in China, which, in turn, increased the prevalence of diseases and harmed the health of China’s residents.

### Environmental pollution impact on human health

The impact of the environment on health mainly depends on the concentration of pollutants, the exposure–response coefficient, and the size of the exposed population in the city. The analytical method based on the exposure–response and the dose–effect principle mainly focuses on research on the loss of health of residents caused by environmental pollution. Matus et al. [38] used a policy prediction model to analyze the fact that the health and economic losses caused by China’s air pollution emissions have caused a substantial burden on the Chinese economy. Chen et al. [39] studied the impact of PM10 on the health of residents in 113 major cities in China, and the results showed that PM10 has caused greater health loss in urban residents in China. In view of the frequent occurrence of the haze phenomenon and the high concentration of PM2.5 in China, Voorhees et al. [40] analyzed the human health loss of Shanghai residents caused by PM and showed that the monetary value of all the deaths that could be avoided if Shanghai’s air quality met the national secondary standards is CNY 1.7 billion to 12 billion. Pearce and Warford [41] pointed out that the most direct consequence of environmental degradation is to jeopardize human health through various physical or mental illnesses and even premature death. In addition, the degree of harm to human health from environmental pollution depends on the level of exposure to pollution, and long-term exposure to heavily polluted areas is more likely to cause disease [42]. Chen et al. [43] found that the heating policy based on the dividing line between the Qinling Mountains and the Huaihe River increased total pollution and shortened the expected life expectancy of residents in the northern region. Cheung et al. [44] constructed instrumental variables based on wind direction and air pollution in the surrounding area and found that air pollution increases the mortality rate of the population.

### Research review

It can be concluded from the above literature that although the current theoretical research and methodologies regarding environmental pollution and trade openness impacting human health have achieved some results, with these results and methods providing better policy guidance for improving human health, this area still requires further improvement and development. First, most of the research methods are based on scenario simulation, and empirical studies are limited by factors such as data availability and external conditions. Scenario simulation is based on hypothetical conditions, and the influencing factors are very limited and deviate from the real situation; this needs to be supplemented by empirical studies. Second, there are fewer theoretical approaches applied to environmental pollution and human health, and the theoretical system is not complete enough, so it is necessary to continue to deepen the research and improve the integrated research model of environmental pollution and human health. Third, existing research is limited to the relationship between the two variables; trade openness can affect public health and also cause environmental pollution problems, so it is necessary to consider the three aspects within the same framework to carry out research more comprehensively and systematically than the impact of a single variable on health. This can be derived from the different directions and degrees of impact, which can provide a solution to the complex relationship between trade, the environment, and the health of the population. We can then realize the construction of a healthy, harmonious, fair, and efficient social environment to achieve the path forward. This can provide a reference for solving the complex relationship between trade, environment, and people’s health. Based on the theoretical foundation of international trade, health economics, environmental economics, and other related disciplines, as well as the research results of related scholars from home and abroad, this research tries to analyze the impact of environmental pollution and trade openness on the human health of China’s residents in a more systematic way, which is a supplement to and improvement of existing research.

## Methodology

### Empirical design

The health economics community has carried out more research on the factors that influence health, among which Grossman’s health production function is the most classic. Grossman [45] established a health demand model as follows:


$$\text{H}=\text{F}\left(\text{X}\right)$$ (1)

where H denotes population health, and X denotes the factors affecting health, such as education, economy, medical conditions, etc. Filmer and Pritchett [46] developed a new health production function on the basis of Grossman’s health production function, and set the model with reference to the new health production function:


$$HH_{it}=\alpha_{it}+\beta_{1}TO_{it}+\beta_{2}EP_{it}+\gamma_{i}Z_{it}+\varepsilon_{it}$$ (2)

where $HH_{it}$ denotes human health in period t of region i, $TO_{it}$denotes the openness in period t of region i, $EP_{it}$denotes the environmental pollution in period t of region i, and $Z_{it}$ is the control variable; $\alpha_{it}$is the intercept term, and $\varepsilon_{it}$ is the random perturbation term. In order to transform the nonlinear relationship between each explanatory variable and human health into a linear relationship, and to smooth the series to reduce the effect of heteroskedasticity on the regression results, the natural logarithmic treatment is carried out for each variable, which results in the following equation:


$$lnHH_{it}=\alpha_{it}+\beta_{1}lnTO_{it}+\beta_{2}lnEP_{it}+\gamma_{i}lnZ_{it}+\varepsilon_{it}$$ (3)

### Variable selection

#### (1) Explained variables.

Human health (HH): Usually, the health of the population is a multidimensional concept; the previous literature used indicators such as population mortality, population life expectancy, neonatal mortality, and maternal mortality to measure HH. Chen and Deng [47] thought that HH includes both quantitative and qualitative dimensions. This work draws on the approach of Chang and Zhong [48] to measure the level of HH by using the population mortality rate, which can reflect the quantity of human health to a certain extent.

#### (2) Explanatory variables.

Trade openness: Existing studies generally agree that trade openness improves human health for a number of reasons: first, trade openness causes domestic residents to consume more foreign products, bringing in healthy food, medical equipment, etc., increasing nutritional intake and reducing morbidity. Second, trade liberalization leads to technological spillovers that promote medical innovation and improve medical efficiency. Some scholars have also found that the negative impact of trade openness on human health is mainly reflected in the population’s nutrition, dietary habits, food safety, and the spread of disease; the expanding scale of foreign trade creates a greater health risk that can, to a certain extent, trigger epidemics, leading to the spread of disease. This paper draws on the practice of Lin [37] to construct the effective tariff rate for imported intermediary goods at the enterprise level. The corresponding calculation formula is the following:


$${\text{TO}}_{it}=\underset{p�\tau_{it}}{\sum }\alpha_{ipt}\times \phi_{pt}$$ (4)

where $\tau_{it}$ is the set of products imported, $\alpha_{ipt}$is the proportion of the amount of import product, p, imported, i, in year t to the total import amount, and $\phi_{pt}$ is the import tariff rate of product p in year t at the level of HS6 code. By weighting the tariff rates of the intermediate inputs imported by enterprises, the effective tariff rate faced by each exporting enterprise can be obtained.

Environmental Pollution (EP): For the measurement of the degree of environmental pollution, most scholars believe that PM2.5, PM10, and other solid particulate matter are the main sources of pollution that affect the quality of the environment and harm human health; however, in reality, this type of data is not easy to obtain. This paper refers to a previous study selected from different areas of industrial sulfur dioxide emissions to characterize the quality of the environment of a region; sulfur dioxide emissions caused the expansion of acid rain, reduced yields of food and cash crops, caused the corrosion and destruction of building materials, and caused the population mortality rate to increase [49–52]. The main reasons for choosing sulfur dioxide emissions are as follows: First, compared with other environmental factors, the exogeneity of the air and exogenous determinants are stronger, which can reduce the endogeneity of this paper; second, the probability of regional residents being exposed to the atmospheric environment is relatively high, which can effectively deal with the shortcomings of the use of macroscopic data; third, the main component of solid particulate matter, such as PM2.5 and PM10, that is, industrial sulfur dioxide, as an indicator of environmental quality, is also verified and recognized by many scholars.

#### (3) Control variables.

Public health expenditure (PHE): More public health expenditure can drive the flow of funds to medical institutions, improving human health. By using survey statistics from Mexico, Mays and Smith [53] found that 48.49% of patients had been unable to get healthcare services in time due to transportation barriers; effective intervention in community transportation and improved healthcare services were suggested in favor of the treatment of patients’ diseases. Through the establishment of an error correction model, the analysis of Akinkugbe and Mohanoe [54] proved that increasing the number of doctors can contribute to the improvement of public health status. Derose et al. [55] argued that public health organizations can contribute to human health improvement by enhancing the capacity of healthcare workers. The public health expenditure in the work refers to the government-budgeted health expenditure in each region, which is the allocated expenditure in the field of healthcare at all levels of government. It is measured by using the per capita government budgeted health expenditure in each region in the “China Health Statistics Yearbook”.

Environmental regulation (ER): As a highly representative measure in the course of China’s air pollution control, the environmental regulation policy has set specific emission reduction targets for the concentration of respirable particulate matter (PM10) and the concentration of fine particulate matter (PM2.5) and has identified 10 specific measures for the realization of the above targets, such as intensifying comprehensive treatment. The overall control of pollution has achieved remarkable results. Specific measures have been taken, and comprehensive pollution control has achieved remarkable results. On the one hand, environmental regulations can effectively reduce the concentration of pollutants such as PM10 and PM2.5; on the other hand, a reduction in pollutant concentration can reduce the number of deaths, mortality rate, and number of premature deaths to a certain extent. Therefore, this paper argues that environmental regulatory policies can, to some extent, improve human health by controlling the pollutants that are harmful to human health. The research uses the completed investment in industrial pollution control as a share of the value added of the secondary industry [56].

Urbanization level (UR): The urbanization rate is generally used to measure the level of urbanization, i.e., the proportion of the urban population in a region to the total population, which is the most basic method to measure the progress of urbanization in countries around the world and is also an important symbol of the degree of economic development of a region [57]. Generally speaking, health, the environment, education, and other public infrastructure and public services in urban areas are better than those in rural areas, and long-term residents of urban areas not only have a more convenient lifestyle but also enjoy faster medical services and a better medical environment in the event of illness, disability, and other unhealthy conditions. Therefore, there is an inextricable link between urbanization and human health. The data on the urbanization rate come from the statistical yearbooks of each province.

Population Size (PS): The population size of a region has a significant impact on regional economic development, resource utilization, and the healthcare environment and ultimately affects the quality of life and health of residents. When a region is overpopulated, the burden on local resources will increase, the human health environment will deteriorate, and the living environment and health of residents will be adversely affected. Moderate population size can promote the smooth development of society and gradually improve the living environment and health of residents. Therefore, in this paper, the total population was chosen to measure the population size of each province, and the data were logarithmically processed from the China Statistical Yearbook, the China Population and Employment Statistical Yearbook, and the statistical yearbooks of each province.

### Regression method: GMM estimation

There is a certain degree of inertia in human health, i.e., prior health conditions may have some influence on current health. The static panel data model does not take into account the health status of the previous period, and by considering the possible endogeneity between variables, we used the dynamic panel data model to verify the variables’ impact on human health. Meanwhile, considering that the ordinary standard error estimation is not accurate, the generalized moment estimation (GMM) is an effective method to overcome the endogeneity problem. Considering the limitations of the short panel data and the possible endogeneity problem that can bias the model estimation, the regression was conducted using the generalized moment estimation method. The generalized moment estimation includes two methods: system GMM and differential GMM. The differential GMM estimation method may have the problem of weak instrumental variables, and the system GMM method can more fully utilize the variable information; considering this, we selected system GMM to empirically analyze the panel data. According to the selection of the variables and methods, the theoretical model is constructed as follows:


$$lnHH_{it}=\alpha_{it}+\beta_{1}lnHH_{it-1}+\beta_{2}lnTO_{it}+\beta_{3}lnEP_{it}+\gamma_{i}lnPHE_{it}+\gamma_{i}lnER_{it}+\gamma_{i}lnUR_{it}+\gamma_{i}lnPS_{it}+\varepsilon_{it}$$ (5)

where $HH_{it}$ denotes human health, $TO_{it}$denotes trade openness, $EP_{it}$denotes environmental pollution, $PHE_{it}$denotes public health expenditure, $ER_{it}$denotes environmental regulation, $UR_{it}$denotes urbanization, $PS_{it}$denotes population size, $\alpha_{it}$is the intercept term, and $\varepsilon_{it}$denotes the random disturbance term.

## Results

### Cross-sectional dependence tests

Cross-sectional dependence is a critical issue when examining the relationship between all selected variables in a panel data model, and ignoring it may lead to serious estimation bias and size distortion. Therefore, before analyzing the smoothness of the variables, the authors first tested for the presence of cross-sectional dependence in the panel and used the Breusch–Pagan LM test and the Pesaran CD test. The results of the tests showed that the p-values were greater than 0.01 (see Table 1), proving that the cross-sectional correlation of the variables was not significant and that there was no cross-sectional correlation in the data.

**Table 1:** Cross-sectional dependence test result.

Variable	BP-LM test	Pesaran CD test
**lnHH**	22.13(0.23)	3.98(0.44)
**lnTO**	44.09(0.34)	4.28(0.11)
**lnEP**	34.87(0.15)	10.27(0.19)
**lnPHE**	77.02(0.26)	7.76(0.64)
**lnER**	10.98(0.44)	6.09(0.34)
**lnUR**	21.48(0.32)	7.98(0.27)
**lnPS**	66.37(0.12)	9.99(0.16)

The values in parentheses are p-values.

### Unit root test

If the variable data are in a nonstationary series, the direct model estimation will show pseudo-regression results and further empirical analysis will result in false conclusions. Therefore, we adopt the ADF statistic to conduct the unit root test on the inter-provincial panel data results, as per Table 2. Under the three types of assumptions of the intercept term and the trend term, the ADF test results of the original value series fail to reject the original hypothesis of the existence of a unit root and are nonstationary. The first-order differenced series all passed the ADF test under the first two types of conditions, indicating that the differenced series is smooth, and therefore, the variables are first-order single-integrated series and can be analyzed in regression.

**Table 2:** Unit root test results.

Variable	Intercept term		Intercept and trend terms		No intercept and no trend terms	
**Original Value Sequence**	**first-order difference**	**Original Value Sequence**	**first-order difference**	**Original Value Sequence**	**first-order difference**
**lnHH**	99.36***	293.93***	121.45***	198.73***	33.74	159.03***
**lnTO**	102.34***	95.33***	134.09***	156.93***	12.38	145.83***
**lnEP**	93.92***	77.94***	19.73	89.64**	82.03**	197.75***
**lnPHE**	81.02**	210.82***	122.93***	188.45***	9.88	99.83***
**lnER**	4.73	234.57***	163.23***	198.37***	55.73	119.47***
**lnUR**	98.91***	318.83***	81.92**	166.83***	21.83	121.43***
**lnPS**	99.49***	239.67***	118.75***	167.22***	12.09	117.99***

* P < 0.1; **P < 0.05; ***P < 0.01.

### Co-integration test

In this paper, Pedroni and Kao’s tests were used to conduct co-integration tests on the data taken, as per the results in Table 3; the p-value of each test method is less than 0.05. The data can pass the co-integration test, and the panel data model can be established for further analysis.

**Table 3:** Co-integration test results.

	Test Methods	Statistic	Prob.
**Pedroni**	Panel PP- Statistic	-55.9321	0.0000
	Panel ADF- Statistic	-15.9943	0.0000
	Group PP- Statistic	-66.2832	0.0000
	Group ADF- Statistic	-14.5563	0.0000
**Kao**	ADF	4.9832	0.0001

### Multicollinearity test

The VIF value of each variable is calculated to determine whether there is a problem of covariance among the variables. If VIF is greater than 10, this means multicollinearity exists. The results in Table 4 show that there is no multicollinearity between the variables.

**Table 4:** Multicollinearity test results.

	lnHH	lnTO	lnEP	lnPHE	lnER	lnUR	lnPS
**VIF**	1.34	2.45	1.99	3.45	0.49	1.23	2.22

### Model type discrimination

Panel data regression equations, including the constant coefficient model without individual effects, the variable intercept equation, the variable coefficient equation, and the intercept term of the variable intercept equation, can be categorized into fixed effects and random effects. Before the panel data regression analysis, it is necessary to determine the type of regression equation and the type of intercept term. An F-test was used for the values in Table 5; the p-value is less than 0.05, so the developed individual fixed effect model is appropriate. Further, in order to discriminate whether the model is an individual random effect model or an individual fixed effect model, the Hausman test is introduced, and the p-value is less than 0.05, as shown in Table 5. Therefore, the original hypothesis is rejected, i.e., the established individual fixed effect model is appropriate. In addition, the object of the study is the mortality rate; there is no phenomenon of random sampling on the whole, so the establishment of an individual fixed effect model is more appropriate.

**Table 5:** Panel data model type discrimination test results.

Test Methods	Full sample	Eastern Region	Central Region	Western Region
**F test**	25.37(0.0000)	77.76(0.0000)	22.12(0.0000)	17.99(0.0000)
**Hausman test**	28.93(0.0000)	14.39(0.0001)	12.32(0.0000)	16.61(0.0002)
**Verdict**	Fixed effect	Fixed effect	Fixed effect	Fixed effect

p-values in parentheses.

### Analysis of regression results

In this paper, the GMM model was constructed using panel data with the help of Stata2017 software. The GMM model is divided into differential GMM and systematic GMM, in which the systematic GMM overcomes some missing errors in the differential GMM. The results can be seen in Table 6 below.

**Table 6:** Regression results.

Variables	National	East	Central	West
**lnTO**	0.1327	−0.2180***	0.0943	0.0896***
**lnEP**	0.0754***	0.0217	0.1371**	0.2721***
**lnPHE**	−0.1350 *	−0.1987 *	−0.1231***	−0.0122***
**lnER**	−0.066***	−0.1562***	−0.1031***	−0.0674 *
**lnUR**	−0.1156***	−0.1289***	v0.0995	−0.1654 *
**lnPS**	−0.1092 *	−0.1168**	−0.1034 *	0.1187 *
**Hasen test**	0.967	0.993	0.834	0.999
**AR(1)**	0.001	0.000	0.000	0.002
**AR(2)**	0.231	0.342	0.453	0.155

Hasen test, AR(1), and AR(2) values are p-values. Note: * *P* < 0.1; E**P < 0.05; ***P < 0.01.

In Table 6, the results of the Hansen over-identification test show that the p-value is greater than 0.05, indicating that the instrumental variables are selected effectively. The results of the AR root test show that the *P*-value of AR (2) is greater than 0.05, indicating that the model does not have more than second-order serial autocorrelation. The above results show that the current model is well constructed.

#### (1) Analysis of trade openness on the impact on human health.

The inhibiting effect of trade openness is mainly reflected in residents’ nutrition, dietary habits, food safety, and the spread of diseases, and the expanding scale of foreign trade has a greater health risk, which can trigger epidemics and lead to the spread of diseases. As the degree of openness to foreign trade has increased, the intake of animal products and non-healthy foods has exceeded moderate levels in many countries, and obesity and non-communicable diseases associated with over-nutrition have emerged. However, trade openness can also improve human health. For example, first, the most direct effect of trade openness is that it allows residents to consume more foreign products, which reduces the cost of consumption and increases the variety of consumption, which, in turn, leads to more healthy food, medical equipment, and so on, which increases nutritional intake, improves hygiene levels, and improves human health. Secondly, trade openness brings technological spillovers, which promote equipment and service innovation in medical institutions and improve medical efficiency; therefore, because the regression results do not pass the significance test, this indicates that the positive effects of trade liberalization have not appeared. This paper concludes that overall trade openness is not significant in relation to public health, which is inconsistent with the conclusions reached by many scholars, such as Liu [58], who used the Bartik method to construct regional export shocks, as well as environmental pollution shocks due to exports, and found that pollution shocks make morbidity rates increase. Yang et al. [59] used panel data from 31 provinces in China from 2005 to 2015, with mediating variables, and applied a variety of environmental indicators; they found that international trade affects the level of national health by exacerbating environmental pollution. The reason for the difference between this paper and previous studies may be in the choice of the data period because, along with economic development, the increase in trade openness brings positive effects that are gradually greater than the inhibitory effects, but this effect has not been fully embodied.

The regression coefficients of the variables are negative in the east and positive in the central and western regions, and they are very significant in the east and west. The possible reason for this is that the eastern region has higher technology, a developed economy, and a higher level of income for residents, who can have more income to buy foreign health food, medical equipment, etc., which can improve human health. The western region, on the other hand, is lagging behind in its level of economic development and has introduced less advanced medical equipment from abroad, while the consumption of high-sugar and high-fat foods, potential food safety risks, and the spread of diseases may have affected a higher proportion of consumption of food, and thus there is a negative effect on the impact on health. The central region is between the eastern and western regions and, to a certain extent, is affected by the spillover effect of the eastern region; thus, the negative effect appears but is not significant. We conclude that the heterogeneity across regions is consistent with the conclusions reached by some scholars, such as Chen et al. [60], who used a panel of 31 provinces and cities from 2000 to 2013 and found that international trade reduces the health of the population through the channel of environmental pollution and that there is a large amount of regional heterogeneity. By using a quasi-experiment on China’s accession to the WTO, Zhang [61] used a double-difference model, where the larger the reduction in import tariffs, the greater the negative impact on health, and this negative impact is more pronounced in regions with lower labor mobility and lower education levels. The difference is that scholars divide the regions differently; most scholars divide by labor or education level or trade openness level; this paper divides the three major regions of East, Central, and West China, comparing the results of the impacts of different regions, which can provide theoretical references for the formulation of corresponding policies in different regions and the co-ordination of balanced development between regions, and is also the innovation of this paper.

#### (2) Analysis of environmental pollution impact on human health.

When looking at the country as a whole, the increase in environmental pollution significantly inhibits human health. This may be because when the social economy grows, energy consumption grows, but the growth in energy consumption will bring a lot of pollution to the environment, and the loss of human health caused by emissions from energy consumption increases with the increase in energy consumption because the consumption of non-clean energy sources, such as coal and oil, releases pollutants such as carbon dioxide, carbon monoxide, sulfur dioxide, and dust particulates, which cause serious environmental pollution problems. Severe environmental pollution, in turn, induces respiratory and cardiovascular diseases, bringing great losses to human health. This is consistent with several other studies; for example, Chen and Chen [5] analyzed the relationship between sulfur dioxide emissions from coal-fired power plants and public health using prefecture-level city panel data as a research sample, and they found that the increase in sulfur dioxide emissions led to a significant increase in the mortality rate of respiratory diseases and the number of lung cancers; Li and Du [62] used the CFPS2012 microscopic survey data to study the relationship between air pollution and residents’ health level; the study found that air pollution has a significant negative effect on residents’ health level. This shows that the conclusions of this paper are in line with the actual situation in China, and the conclusions can provide a theoretical reference for the exploration of policies.

In different regions, this phenomenon is more obvious in the central and western parts of the country; in the eastern part of the country, although it also reduces the level of human health, it is not significant. However, central and western regions are mainly dominated by the energy industry and rely excessively on the opening and use of the energy industry, thus causing serious negative impacts on the environment, which can not improve human health. Due to its lower level of economic development and high concentration of energy, the western region relies more on energy industry development to promote economic growth, thus having the greatest impact on the environment and health. There is a gap between the research in this paper and some other scholars’ studies; many scholars divide the differences in impacts by demographic structure [63], social class [64], and income level [65], which provides a reference to the research in this paper. A few scholars carry out their research by dividing China into different regions; for example, Qu [66] conducted a regional study when investigating the impact of environmental pollution on the public health level. Their study shows that industrial soot emissions are positively correlated with mortality rates for the whole country as well as the east, central, and western regions; there are also scholars who agree with the conclusions drawn in this paper; for example, Hu [67] found that for the whole country, as well as the central and western regions, environmental pollution has a significant positive effect on the residents’ health expenditure. In the eastern region, the effect of environmental pollution on residents’ health expenditure is not significant. This may be due to the fact that, along with economic development, the eastern region is economically developed, with a high technological level, and is more inclined to use clean energy. The scale of environmental pollution is relatively small, and thus, the negative impact on public health is smaller; at the same time, enterprises actively carry out green innovation, which greatly reduces the level of environmental pollution, and thus the impact on human health is smaller, which also reflects China’s contribution to green development. This study can be used to formulate relevant policies for the region, which can be used to improve the health of the population. The research in this paper can provide a theoretical reference for the development of relevant policies in the region.

#### (3) Control variables.

At the national level, urbanization can significantly improve the human health of residents. This is consistent with the findings of Chen and Deng [47]. It shows that although urbanization has a positive and negative impact on human health, overall, urbanization improves human health. When looking at different regions, urbanization can decrease population mortality in the eastern region, indicating that the increase in urbanization has a positive role in improving human health; in the central region, urbanization is not significant; and in the western region, although urbanization can decrease the population mortality rate, the significance is relatively low. The possible reasons for their different economic foundation are as follows: the eastern regions have better human health services and a higher level of resident human capital, and the proportion of the tertiary industry is becoming higher and higher; however, in the central and western regions, the secondary industry still occupies a large proportion of the three industries, and environmental issues are important. In addition, the urban-oriented development policy has also made the urban–rural gap in the central and western regions obvious, so the negative effect of urbanization development on the human health of the residents is obvious.

Public health expenditures can significantly decrease the population mortality rate, indicating that public health expenditures’ impact on human health is positive. Increasing public health expenditures is conducive to reducing the overall population mortality rate, and the coefficient of the impact is negative in all regions, yet the elasticity coefficient in the western region is small, indicating that public health expenditures in the western region have also been lowered and that further increases in the level of expenditure are needed to improve human health.

The population size is not conducive to the improvement of human healthcare at the national level, probably because it increases the social burden faced by a region, exerting great pressure on the construction of local public facilities and human health environments, which, in turn, affects the pace of development of the local economy, the quality of life of the residents, and, ultimately, jeopardizes the health status of the residents. The increase in population size means an increase in the number of people in the region. In cities with a high population size, close external exchanges, frequent exchanges of people, and more emissions from the “three wastes” of production and life increase the risk of infectious diseases breeding and spreading and the pressure on medical supplies, posing a greater challenge to the governance of human health in the city. This poses a greater challenge to human health governance. The test results for the central and western regions show that the growth process of population density must be accompanied by the continuous enhancement of urban medical resource supply capacity, basic public service guarantee capacity, and comprehensive urban health management capacity, and if the corresponding capacity lags behind the population growth, a negative health effect of population concentration will dominate, which matches the findings of Chen et al. [5]; thus, an increase in population will reduce the level of human health. However, according to the theory of the agglomeration effect, in cities with higher population density, there may also be an agglomeration economy and increasing returns that scale in human health resources and infrastructure inputs. The average cost of monitoring and controlling human health risks in cities is relatively inexpensive, and the human health incident disposal plan is flexible and complete. It is also more convenient to utilize the city’s human capital effect, which can obtain a higher level of health outputs, and the results of the tests at the national level for the eastern and central regions are consistent with the findings of Chen et al. [5]. This is confirmed by the results of tests in the eastern and central regions, and similar conclusions are obtained by Yu et al. [17].

Environmental regulation decreases the mortality rate of the population and, thus, improves human health at the national level, mainly because environmental regulation can effectively reduce the concentration of pollutants such as respirable particulate matter (PM10) and fine particulate matter (PM2.5). On the other hand, the reduction in pollutant concentration can reduce the number of deaths, mortality rate, and the number of premature deaths to a certain extent. Meanwhile, in the cleaner production audit, the environmental one-vote veto system ensures environmentally friendly economic production methods starting with the source of economic production methods, and environmental regulation increases the cost of environmental pollution in foreign trade, which helps to force enterprises to innovate green production processes. From the perspective of different regions, the effect of the eastern region is the largest, and the effect of the western region is the smallest, which is mainly related to the regional economic growth mode; in the western region, the resource industry is dominated by the implementation of environmental regulation policy that is often not strict enough, affecting policy effectiveness and, thus, inhibiting human health.

### Robustness testing

The method of robustness testing, which involves a data substitution method, a variable substitution method, setting control or dummy variables, and so on, does not exist singularly. In this paper, according to the model and data situation, by transforming the explanatory variables, PM2.5 was selected to replace industrial SO2 re-estimation in order to avoid the problem of obtaining accidental results due to the use of specific pollution indicators. The results of the robustness test can be seen in Table 7. After the test, the direction and significance of the regression coefficients of the variables did not particularly change, and the size of the regression coefficient value did not change much. It further confirms the results of our empirical regression earlier.

**Table 7:** Robustness test results.

Variables	National	East	Central	West
**lnTO**	0.1123	−0.2009***	0.0765	0.0567***
**lnPM**	0.1032***	0.0445	0.1618**	0.2657**
**lnPHE**	−0.1127 *	−0.1678 *	−0.1318***	−0.0318***
**lnER**	−0.0809***	−0.1785***	−0.1218***	−0.0504 *
**lnUR**	−0.1124***	−0.1346***	−0.0804	−0.1994 *
**lnPS**	0.1145**	−0.1349**	0.1097***	0.1218**
**Hasen test**	0.945	0.978	0.904	0.983
**AR(1)**	0.000	0.000	0.000	0.000
**AR(2)**	0.279	0.311	0.245	0.132

* P < 0.1; **P < 0.05; ***P < 0.01.

## Conclusion and implications

### Conclusion

We used the inter-provincial panel macro data of 30 regions to study the impact of trade openness and energy consumption on human health and drew the following conclusions:

The negative impact of trade openness on human health is reflected in the fact that trade openness may affect the nutrition of the population, their dietary habits, food safety, and the spread of diseases, but it can also bring more healthy food and medical equipment, increase nutritional intake, improve the level of hygiene, and improve human health. At the national level, the impact of trade openness on human health is not significant. From the perspective of different regions, trade openness can improve the level of human health in the eastern region but is unfavorable to the level of human health in the central and western regions.

The consumption of unclean energy sources, such as coal and oil, releases pollutants such as carbon dioxide, carbon monoxide, sulfur dioxide, and dust particulate matter, causing serious air pollution problems. Serious air pollution induces respiratory diseases and cardiovascular diseases, bringing huge losses to human health. From the results of the empirical analysis, environmental pollution reduces human health in all regions. However, the negative effect is not significant in the eastern region, which is related to the high proportion of clean energy use in the eastern region, while the central and western regions mainly have the energy industry as their dominant industry and are overly dependent on the openness and use of the energy industry, thus causing serious negative impacts on the environment, which is not conducive to the improvement of human health.

Urbanization can reduce the national population mortality rate and improve human health, and from the perspective of different regions, the coefficient of the impact of urbanization on the population mortality rate in the eastern region is significantly negative. This is consistent with the estimation results at the national level. In the central region, the coefficient of urbanization’s impact on the population mortality rate is negative, but none of them passes the significance test; in the western region, the coefficient of urbanization’s impact on the population mortality rate is negative but has a lower significance level. Increases in human health expenditures reduce population mortality and have negative coefficients in all regions. Increasing population size is not conducive to improving urban human health at the national level in the west and central regions, but it can improve the health of the population in the east. Environmental regulation policies are effective in reducing the national population mortality rate and, thus, enhancing human health, with the largest effect in the eastern region and the smallest effect in the western region, mainly due to a strong correlation with regional economic growth patterns.

### Recommendations

Based on the results of the empirical analysis above, trade openness and energy consumption both have a greater impact on human health, and there is regional heterogeneity. This paper makes recommendations to improve human health using the following methods:

(1) First, make full use of the technological spillover effects of trade openness to improve medical care and reduce medical costs. Second, trade openness brings environmental pollution at the same time, which offsets part of the health effects and increases healthcare expenditures. In the process of deepening trade openness, we should pay attention to pollution prevention and control and emphasize the co-ordination and matching of trade and environmental policies to reduce the environmental pollution caused by trade openness and improve the health effect that trade openness is supposed to have.
(2) The conversion rate of coal resources should be the primary goal, and specific management measures should be formulated in conjunction with clean coal technology. Meanwhile, in northern regions with high demand for coal in winter, coal resources should be fully utilized to transform traditional inefficient heating structures and promote the use of clean coal. In addition, for heavy industrial areas, fiscal and tax incentives should be used as a means to guide enterprises to actively innovate, research, and develop new energy technologies, as well as, at the same time, regulate the emission of pollution in order to take effective measures to reward and punish practice. In the process of industrial undertakings, for the promotion of economic development in the central and western regions at the same time, it is necessary to adhere to the principle of energy saving and environmental protection, abandon the serious pollution of the industry, and actively advocate for the benign development of enterprises and healthy development, taking on the task of environmental protection.
(3) Improve the level of environmental regulations in less developed regions and promote the optimization and upgrading of the foreign trade structure in the central and western regions. High-energy-consuming and high-polluting import and export enterprises have chosen to locate in the central and western regions in order to economize on environmental costs, exposing the residents of the central and western regions, who have lower per capita income levels, to higher concentrations of environmental pollution, which has led to environment-related health inequalities. The level of environmental regulation in the central and western regions should be raised, and policy incentives should be provided to import and export enterprises that make innovations in green production technology.
(4) For precise policymaking, as there are large differences in regional endowments and conditions, it is necessary to analyze the actual situation in different regions and take into account the impact of differentiation. When formulating policies, specific issues should be analyzed, and policies should be precisely applied to the actual situation in different regions, abandoning the “one-size-fits-all” phenomenon of health trade policies that existed in the past and promoting synergistic development. In order to improve the health of the population, the eastern region should provide better public services and reduce pollution; the central region should increase government expenditure on public services, vigorously promote urbanization, stabilize employment, improve the efficiency of the use of healthcare resources, and combat pollution; the western region should invest more resources in education and develop rural revitalization to reduce the gap between urban and rural areas.
(5) The importance of health expenditures should be emphasized in fiscal expenditures. Among the various financial expenditures, health expenditure and education expenditure are expenditure items that prove difficult in terms of bringing economic utility and financial revenue to the government in the short term; some local governments, in pursuit of the performance view, tend to pay more attention to the items that can rapidly develop the economy and increase financial revenue and do not pay enough attention to the field of health. Therefore, it is necessary for governments at all levels to further emphasize the importance of health expenditures in fiscal expenditures and to maintain stable growth of human health expenditures in tandem with economic development and fiscal expenditures.

### Shortcomings and prospects

(1) As both environmental pollution and trade openness may have spatial spillover effects, which, in turn, have an impact on the public health of the neighboring regions, the aim of this paper was to develop spatial research, which may bias the actual results; this could provide a direction for future research in this area;
(2) For the selection of variables, due to the fact that there are a lot of factors that affect the level of health, this paper does not list all of the influencing factors, referring only to previous scholars’ research to select some key factors to carry out the study. In future research, we will explore further influencing factor analysis and strive to draw different conclusions;
(3) In this paper, we did not test the nonlinear and threshold effects, and we will further explore the nonlinear relationship and threshold value in future research so as to provide a clearer theoretical reference for the region to formulate relevant policies.
